# Computer-Aided Quantification of Interstitial Lung Disease from High Resolution Computed Tomography Images in Systemic Sclerosis: Correlation with Visual Reader-Based Score and Physiologic Tests

**DOI:** 10.1155/2015/834262

**Published:** 2015-01-05

**Authors:** Fausto Salaffi, Marina Carotti, Silvia Bosello, Alessandro Ciapetti, Marwin Gutierrez, Elisabetta Bichisecchi, Gianmarco Giuseppetti, Gianfranco Ferraccioli

**Affiliations:** ^1^Department of Rheumatology, Polytechnic University of the Marche, Ancona, Italy; ^2^Department of Radiology, Polytechnic University of the Marche, Ancona, Italy; ^3^Division of Rheumatology, School of Medicine, Catholic University of the Sacred Heart, Rome, Italy

## Abstract

*Objective.* To evaluate the performance of a computerized-aided method (CaM) for quantification of interstitial lung disease (ILD) in patients with systemic sclerosis and to determine its correlation with the conventional visual reader-based score (CoVR) and the pulmonary function tests (PFTs). *Methods.* Seventy-nine patients were enrolled. All patients underwent chest high resolution computed tomography (HRCT) scored by two radiologists adopting the CoVR. All HRCT images were then analysed by a CaM using a DICOM software. The relationships among the lung segmentation analysis, the readers, and the PFTs results were calculated using linear regression analysis and Pearson's correlation. Receiver operating curve analysis was performed for determination of CaM extent threshold. *Results.* A strong correlation between CaM and CoVR was observed (*P* < 0.0001). The CaM showed a significant negative correlation with forced vital capacity (FVC) (*P* < 0.0001) and the single breath carbon monoxide diffusing capacity of the lung (DLco) (*P* < 0.0001). A CaM optimal extent threshold of 20% represented the best compromise between sensitivity (75.6%) and specificity (97.4%). *Conclusions.* CaM quantification of SSc-ILD can be useful in the assessment of extent of lung disease and may provide reliable tool in daily clinical practice and clinical trials.

## 1. Introduction

Systemic sclerosis (SSc) is a heterogeneous autoimmune disorder of unknown aetiology that is characterized by musculoskeletal involvement, vascular dysfunction, cutaneous and visceral fibrosis [1]. Interstitial lung disease (ILD) was reported in up to 70% of patients with SSc and frequently it can be cause of death of these patients [1–3].

High resolution computed tomography (HRCT) is currently the most accepted imaging tool for the detection, characterization, and treatment monitoring of ILD [3–7]. Moreover its findings have demonstrated a good correlation with the pulmonary function tests (PFTs) acquiring a prognostic value for ILD [4, 8].

Despite these characteristics the correct interpretation of HRCT findings still represents often a problem for the inexperienced physicians since there is a wide interobserver variability even among expert radiologists [9]. Therefore, a quantitative, noninvasive and reliable imaging method able to permit an accurate assessment of ILD in SSc is highly desirable [10, 11].

To date, several computerized tools to segment automatically the lung, using HRCT images, have been developed [12]. They include image display (e.g., multiplanar reformations and surface shading for three-dimensional and volume rendering), anatomic image quantitation (e.g., area and volume of airways and lungs), and regional characterization of lung tissue (analyzing attenuation, changes in attenuation, and texture patterns in the imaged lung) [12–14]. They also provide computer-derived measures such as mean lung attenuation (MLA) (representing the average global attenuation value of the pulmonary parenchyma), skewness (representing the extent of asymmetry of histograms), and kurtosis (representing the degree of “peakedness” of the histograms) [15]. Additionally, the acquisition of more sophisticated image analysis including the fractal analysis and the adaptive multiple feature method is possible [16].

With respect to the traditional visual interpretation of HRCT lung findings, the automatic computer-based assessment may improve the objectivity, sensitivity, and repeatability of quantitative changes in the lung features. We recently investigated the utility of an open-source Digital Imaging and Communication in Medicine viewer software OsiriX to assess ILD in patients with SSc showing a significant association between the quantitative OsiriX assessment and the conventional HRCT semiquantitative analysis. Results for the reliability of the open-source findings were also acceptable [17].

Taking into account this information we designed the present study aimed to evaluate the performance of a computerized-aided method (CaM) for the quantification of ILD, in patients with SSc and to determine its correlation with respect to both the conventional visual reader-based score (CoVR) and the PFTs findings. The secondary aims were to evaluate the feasibility and interreader reliability of the CaM.

## 2. Materials and Methods

### 2.1. Patients

Patients with SSc, defined by the American College of Rheumatology (formerly, the American Rheumatism Association) classification criteria [18], were included in the study. SSc patients were classified in limited and diffuse cutaneous involvement (lcSSc and dcSSc, resp.). LcSSc was characterized by thickening of the skin distal to the elbows and knees and proximal to the clavicles (including the face) whereas dcSSc was characterized by thickening of the skin proximal as well as distal to the elbows and knees and including the trunk and the face. Exclusion criteria included absence of recent or current respiratory infection, severe pulmonary hypertension requiring specific treatment, uncontrolled congestive heart failure, known history of asthma, allergic alveolitis, and exposure to organic dusts or clinically significant abnormalities other than interstitial lung disease identified on chest radiography or on HRCT.

### 2.2. Pulmonary Function Tests

PFTs were performed within 1 week from the lung HRCT assessment by a flow-sensing spirometer and a body plethysmograph connected to a computer for data analysis. PFTs were performed while the patient was at rest in a seated position. These tests consisted of spirometry using a computerised lung analyser (*MasterScreen Diffusion, Jaeger GmbH, Höchber, Germany*). Forced vital capacity (FVC), forced expiratory volume in 1 s (FEV1), and the single breath carbon monoxide diffusing capacity of the lung (DLco) were obtained. These parameters of PFT were expressed as percentage of predicted value. At least three measurements were taken for each variable to guarantee repeatability.

### 2.3. HRCT Assessment and Visual Reader-Based Disease Quantification

All HRCT examinations were performed according to standard protocol using a CT 64 GE light Speed VCT power scanner with a rotation tube scanning time of 0.65 s. Scans were obtained at full inspiration from the apex to the lung base with the patients in the supine position, at 120 kV and 300 mAs and slice thickness and spacing of scans of 1.25 mm and 7 mm, respectively. HRCT assessment did not include the use of contrast media agents. The parenchymal abnormalities on HRCT were coded and scored in all the images by two independent readers, blinded with respect to the results, according to Warrick et al. [11]. A point value was assigned to each abnormality as follows: ground-glass appearance = 1; irregular pleural margins = 2; septal/subpleural lines = 3; honeycombing = 4; subpleural cysts = 5. In each patient the “severity of disease” score was obtained by adding single point values. The mean values of the two independent readers were used as a final control group. An “extent of disease” score was obtained by counting the number of bronchopulmonary segments involved for each abnormality: one to three segments scored as 1; four to nine segments scored as 2; more than nine segments scored as 3. The severity and extent of disease were then calculated as total HRCT score (range from 0 to 30). The HRCT examinations were randomised and reviewed by two radiologists (E.B and M.C) with more than 15 years of experience in general and thoracic radiology who were unaware of clinical or functional findings. The preliminary agreement between the two radiologists with regard the total HRCT scores was good: intraclass correlation coefficients (ICC) 0.81.

### 2.4. Computerized-Aided Scoring Quantification Process

**Figure 1:**
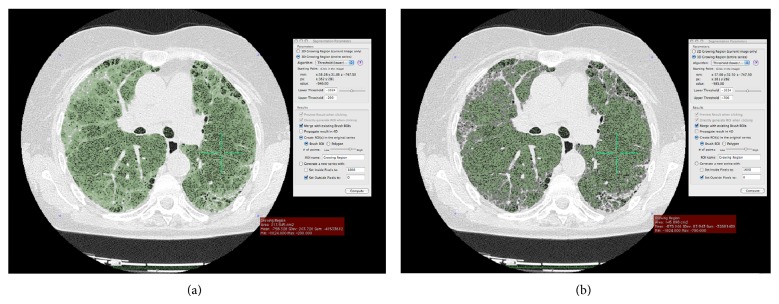
Representative sequences of the OsiriX segmentation process. We have developed the pulmonary fibrosis fraction by the following formula: total HRT lung volume (−1.024 to −200) – nonfibrotic HRCT lung volume (−1.024 to –700) divided by total HRT lung volume (−1.024 to −200) multiplied by 100.

HRCT images were reconstructed and analysed by OsiriX, a DICOM viewer software [19] (OsiriX version 3.9; Apple Computer) on a Mac Mini (2.8 GHz Intel Core 2 Duo Desktop Computer, 16 GB random-access memory; Apple Computer, Cupertino, CA, USA) running Mac Operating System X 10.8.5. After inserting the DVD containing HRCT data in the drive, the DICOM data were automatically extracted from the disc by OsiriX. The DICOM data were stored in the OsiriX using the ‘‘Copy linked files to Database folder” under ‘‘file” in the OsiriX dropdown menu. The program uses a semiautomated thresholding technique to isolate the lungs from other tissues and structures. For each section, a semiautomatic lung parenchymal segmentation was performed in order to obtain analysis of all images (Figure 1). Then, descriptive parameters of the computer analysis were calculated. The radiodensity of the lung parenchyma isolated from the mediastinum and the thoracic wall ranges between −200 and −1024. According to Shin et al. [20], the value of radiodensity for ILD was considered from −700 to −500. So, in the present study, the thresholds of −1024 and −700 were used for the evaluation of the nonfibrotic HRCT lung volume. Adopting these radiodensity values we calculated the pulmonary fibrosis fraction.

Figure 1 illustrates the sequences of the OsiriX segmentation process. A minimal user intervention in the CaM (one author) was required to exclude lung structures not relevant for the assessment (i.e., trachea, blood vessels, and large bronchi near the hilum).

### 2.5. Statistical Analysis

All data were entered into a Microsoft Access database developed for the management of all data. The data were analysed using the SPSS version 11.0 (SPSS Inc., Chicago, IL) and the MedCalc version 10.1 (MedCalc Software, Mariakerke, Belgium). Measurement reproducibility of repeated OsiriX-based assessments and interobserver agreement between the two readers of HRCT were tested using the ICC. This value is an expression of 95% of all measurements that is expected to be included within the range (limits of agreement). Feasibility of computerized analysis by OsiriX was estimated by comparing the time spent for the quantitative analysis using the CaM with respect to HRCT CoVR semiquantitative analysis by the independent samples “*t*” test. The relationships among the lung segmentation analysis, the readers, and the PFTs results were calculated using linear regression analysis and Pearson's product moment correlation (“*r*” values). Student's *t*-test was used to compare two subgroups of the study population for continuous characteristics, and the chi-square test was used for categorical characteristics. Differences corresponding to *P* < 0.05 were considered significant.

Receiver operating curve (ROC) analysis was also performed for determination of CaM optimal extent threshold. ROC curve was plotted to determine the area under the curve (AUC) and determine sensitivity, specificity, positive and negative predictive values, and positive likelihood ratio (LR+). Although there is no official consensus with this regard, fibrotic scores on CoVR, defined according to Warrick et al. [11], were categorised into two groups as follows: ≤7 (mild lung fibrosis) and >7 (severe lung fibrosis). A minimum score of 7 on CoVR system would be required to consider HRCT abnormalities in SSc as predictive of pulmonary disease [21]. We used this cut-off as external criteria to dichotomize the patients. The nonparametric Wilcoxon's signed rank test was used for calculation and comparison of the areas under the ROC curves (AUC-ROCs) derived from the sample of patients.

## 3. Results

### 3.1. Patients

The study group included 79 patients (12 male, 67 female, mean age 59 ± 9.7 years) with SSc. The average disease duration was 9.3 ± 5.8 years. Thirty-eight patients were classified as having dcSSc (mean age, 61 ± 9.6 years; range, 33 to 78 years; disease duration, 10.9 ± 6.3 years) and 41 patients were classified as having lcSSc (mean age, 56 ± 9.2 years; range, 31 to 69 years; disease duration, 8.5 ± 5.9 years). The group of patients having dcSSc, in comparison with lcSSc patients, was older (62.3 ± 8.5 versus 57.1 ± 9.4 years; *P* < 0.05) and with a long-term disease (10.4 ± 5.4 versus 8.6 ± 5.9 years; *P* < 0.05). The mean (±SD) time interval between PFTs and HRCT was 4.5 ± 1.5 days (range: 0–7 days). On PFTs, average FVC was 89.6 ± 9.6% of predicted, average FEV1 was 83.8 ± 8.7% of predicted, and average DLco was 70.1 ± 15.9% of predicted. FEV1 and DLco were statistically different in the two groups of SSc patients (*P* < 0.001). All patients displayed HRCT findings of ILD (detected by the readers: average total HRCT score = 12.1 ± 6.9). Similarly, the percentage of extent of lung diseases measured by CaM was significantly higher, independently of the gender, in patients with dcSSc (22.1 ± 9.7% versus 16.3 ± 9.0; *P* = 0.008).

### 3.2. Correlation between Computerized-Aided Method Results, Visual Reader-Based Scoring Method, and PFTs

**Figure 2:**
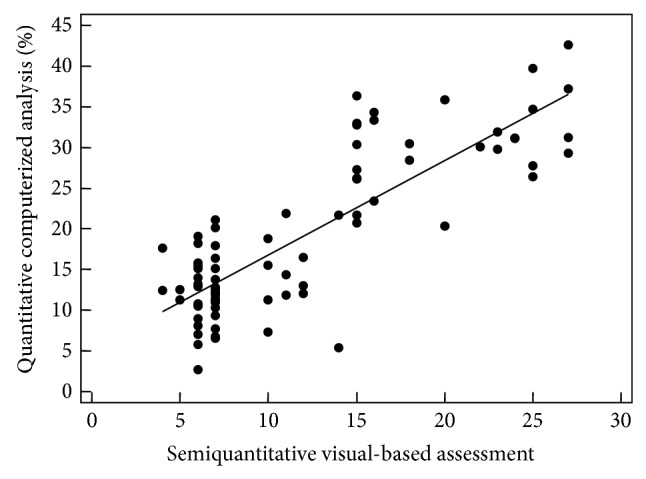
Scatter plots with regression line, illustrating the correlation between computerized-based analysis and visual reader-based scoring method.

**Figure 3:**
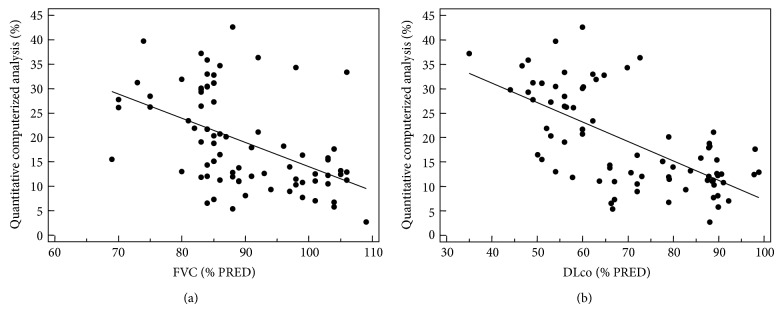
Scatter plots showing correlations between PFTs and quantitative computerized analysis of pulmonary fraction with regression line. Each circle shows a single patient data.

A close correlation between CaM results and CoVR was observed (*r* = 0.829; *P* < 0.0001) (Figure 2). The computerized-aided scores showed a moderate to highly significant negative correlation with forced vital capacity (FVC) (*r* = −0.490; *P* < 0.0001), forced expiratory volume in 1 s (FEV1) (*r* = −0.675; *P* < 0.0001) and the single breath carbon monoxide diffusing capacity of the lung (DLco) (*r* = −0.653; *P* < 0.0001) (Figure 3).

### 3.3. Determination of a CaM Optimal Extent Threshold

**Figure 4:**
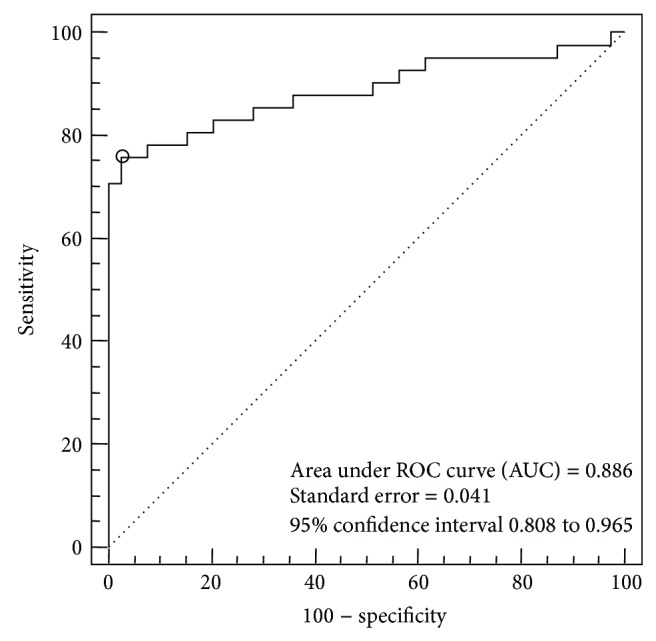
ROC curve for determination of CaM optimal extent threshold. The circle on the curve shows optimal cut-off point, corresponding with the maximum sum of sensitivity and specificity.

Figure 4 shows the ROC curve representation of CaM values for threshold of HRCT findings. The ROC analysis demonstrated excellent performance with an AUC of 0.886 (SE, 0.041) (Figure 4). For the CaM, a cut-off 20% of lung involvement represented the best compromise between sensitivity (75.6%) and specificity (97.4%), with a positive predictive value of 92.2 and a +LR of 29.9. A value of 10% for quantitative lung disease increased the sensitivity to 95.1% but decreased the specificity to 30.7%, whereas a value of 30% increased the specificity to 100% but decreased the sensitivity to 34.1% (Table 1).

### 3.4. Feasibility

The mean time spent completing the quantitative evaluation by CaM was 1.3 min (range 1 to 2.1 min) whereas it was 10.9 min (range 5.9 to 14.9 min) adopting the semiquantitative visual assessment. The difference was highly significant (Student's *t*-test, *P* < 0.0001).

## 4. Discussion

An accurate characterization and quantification of ILD is essential for a correct clinical management of patients with SSc [10, 11, 22]. Our results indicate that the CaM analysed by OsiriX provides a good concurrent validity, reliability, and feasibility for the assessment of ILD in patients with SSc. Considering the promising advent of user friendly software's [19], this approach may be effectively used in both clinical practice and research setting.

To date different visual-based semiquantitative approaches to assess ILD have been proposed [11, 22]. In most of them the final score was calculated either by agreement between two reviewers or by obtaining the mean of the reading scores by two reviewers. Warrick et al. [11] have proposed a semiquantitative method in patients with SSc which allows the evaluation of the different patterns of abnormalities, rated according to the severity and extent of lung damage, through a total overall HRCT score. Kazarooni et al. [22] have divided each lung into three zones (lung apex to aortic arch, aortic arch to inferior pulmonary veins, and inferior pulmonary veins to lung bases) and scored the extent of lung abnormality of each zone on a scale ranging from 0 to 4. More recently, a simplified scoring system based on the grade of the lung involvement more or less than 25% has also been suggested [4].

The CoVR method plays an important role in the interpretation of ILD patterns. Moore et al. [23] have shown that a simple and quick grading system for the extent of total lung disease on HRCT has prognostic significance in SSc, even after adjustment for other prognostic covariates. Similarly, Goh et al. [4] have demonstrated that an easily applicable limited/extensive staging system for SSc-ILD, based on combined evaluation with HRCT and PFTs, provides discriminatory prognostic information. In particular, the risk of death in SSc-ILD and, separately, progression of disease rose strikingly when the overall percentage of lung involved on HRCT exceeded 20% [4]. This threshold value was defined using formal CoVR scoring systems, which is seldom practicable in routine practice.

Although the CoVR is currently the most popular method used [24], it has several disadvantages such as subjectivity and difficulty in estimating accurately the different components of disease (honeycombing, reticular, and linear ground-glass opacity). A further difficulty is represented by the complex task of integrating the extent of the abnormalities seen on several HRCT slices and deriving a quantitative measure of the total extent of abnormality of a lung zone or within the lung. Finally, CoVR scoring systems provide lack of reproducibility, with larger interreader and intrareader variation [9]. Compared with visual-based assessments, CaM scores are demonstrated to improve objectivity, sensitivity, and repeatability when measuring the quantitative changes in ILD [16, 17].

As mentioned before, our preliminary experience using this system in SSc patients showed a high agreement with respect to the semiquantitative HRCT analysis performed by experienced radiologists and a significant association between the descriptive parameters by both the quantitative OsiriX assessment and the HRCT semiquantitative analysis [17]. It has been previously shown that there is a significant variability in the lung density in normal individuals, and this factor should be taken into account when considering the use of CT lung density mapping for the assessment of pulmonary disease. However, the radiodensity of the lung parenchyma isolated from the mediastinum and the thoracic wall ranges between −200 and −1024. CT attenuation of normal lung parenchyma is reported to range from −800 to −900 HU, depending on inspiration or expiration, on the level of inspiration achieved for the scan, and on anatomical location that is ventral or dorsal portion [25]. Shin et al. [20] defined the area with attenuation between −500 and −700 as the value of radiodensity for ILD. The author included both ground-glass opacity and reticular opacity. Contrary Yabuuchi et al. [26] used the thresholds of −500 and −800 HU for the evaluation of ground-glass opacity. Moreover the CT attenuation values for consolidation and ground-glass opacity were separated and the radiodensity of −500 UH was selected as the thresholds between consolidation and ground-glass opacity. However, the application of a threshold value of −800 HU may include small peripheral pulmonary vessels and cause an overestimation of interstitial lung disease [27]. In our method, in agreement with Shin et al. [20], −700 HU is selected as the predefined threshold to obtain lung regions.

Our current results confirmed that the quantitative OsiriX assessment system correlate well with visual-based scoring techniques for the detection of HRCT extent and severity of disease [17]. The percentage of extent of lung disease showed a significant correlation (*P* < 0.0001) with FVC, FEV_1_, and DLco.

In a purely clinical context, we have shown that CaM scoring system of ILD may have several advantages in the management of SSc patients. First, the advantage of the OsiriX-based measurement is the use of a continuous scale, rather than a categorical Likert scale. The continuous computerized scoring, in comparison with categorical rating used in the visual (semiquantification) assessment, provides greater power for detecting a treatment effect within a given sample size or allows an approximately 50% reduction of the sample size [28]. The possibility of having the percentage extent of total lung disease, easily obtainable from the rheumatologist in a clinical outpatient setting with a simple and rapid procedure, represents a clear advantage for the assessment of responsiveness including prognostic value data. Secondly, the OsiriX segmentation algorithm proved to be time-efficient, reproducible, and requiring less than two minutes for the total lung evaluation. A third advantage is that OsiriX-based computerized scoring system can be implemented in the setting of a multicenter trial in SSc-ILD using digitized HRCT images. Finally, OsiriX is user friendly open-source software [17, 19] that even rheumatologists can easily manipulate and generate 3D reconstructed images and acquire whole images of 3D anatomical structures. The training in using OsiriX software can be easily and quickly completed [19], providing clinicians with a valuable tool for the evaluation of disease extent and interpretation of patterns of pulmonary function impairment in SSc patients.

We are aware of some limitations in our study. First, the diagnosis of pulmonary fibrosis was based on radiological findings, not by histological examination. Secondly, the CaM scoring system used in this study focuses on quantification of total disease extent and lacks a differentiation between different radiographic patterns, but the clinical significance of these HRCT features is as yet unknown. However, the disease extent has been shown to be a strong predictor of functional pulmonary impairment. Furthermore, our quantitative evaluation did not focus on anatomic compartments of the lung; however, in comparison to emphysema, ILD tends to be widespread. Formal HRCT scoring, especially in clinical trials, is commonly performed using predefined anatomic levels rather than pulmonary lobes, as HRCT examinations are still widely performed due to radiation protection. Third, the use of our density mask method for the quantitative analysis of ILD could not discriminate accurately the low attenuation areas of honeycombing from the normal lung density when honeycombing cysts are present which may underestimate the ILD severity. Therefore, the discrepancy of quantification between the CaM scoring systems and CoVR method may be intrinsic to the densitometric analysis. Regarding this intrinsic discrepancy, the usefulness of the automated system was criticized due to the usual presence of lung increased density (ground-glass opacities) and decreased density (cystic spaces, honeycombing) [29]. Finally, the sample size of our study was limited and the effect of pulmonary hypertension was not assessed. Therefore there might be a limit in the comparison with measures of disease severity.

In conclusion, our results showed that the CaM using an open-source software DICOM application—OsiriX—may assist the rheumatologist analysis of lung HRCT data and provides an objective method for supplementing subjective visual-based grading of the extent of ILD to achieve precise and reader-independent quantification. Compared with previous in-house software, OsiriX will enable wider use, resulting in easier computer-aided technique application in routine practice and better communication among different hospitals.

Computer-derived extent of total lung disease appears as discriminant method and, therefore, can help to produce an objective measure and to obtain prognostic information in SSc-ILD. Although these encouraging data require further validation in prospective studies, we believe that the CaM may improve the ability of rheumatologists to quantify accurately the extent of ILD in SSc patients in both daily clinical practice and clinical trials.

## Figures and Tables

**Table 1:** Criterion values and coordinates of the ROC curve.

Criterion	Sensitivity	95% CI	Specificity	95% CI	+LR	95% CI	−LR	95% CI	+PV	95% CI	−PV	95% CI
>5.82	97.56	87.1–99.9	5.13	0.6–17.3	1.03	0.9–1.1	0.48	0.04–5.0	65.6	53.9–76.1	53.1	1.6–99.1
>6.52	97.56	87.1–99.9	7.69	1.6–20.9	1.06	1.0–1.2	0.32	0.03–2.9	66.2	54.5–76.7	62.9	8.0–98.7
>7.35	95.12	83.5–99.4	12.82	4.3–27.4	1.09	0.9–1.3	0.38	0.08–1.8	67.0	55.0–77.5	58.6	16.8–92.4
>8.10	95.12	83.5–99.4	17.95	7.5–33.5	1.16	1.0–1.4	0.27	0.06–1.2	68.3	56.3–78.8	66.5	25.1–94.4
>9.36	95.12	83.5–99.4	23.08	11.1–39.3	1.24	1.0–1.5	0.21	0.05–0.9	69.7	57.6–80.0	71.8	32.1–95.7
>10.81	95.12	83.5–99.4	30.77	17.0–47.6	1.37	1.1–1.7	0.16	0.04–0.7	71.8	59.6–82.1	77.3	43.5–96.0
>11.49	92.68	80.1–98.5	43.59	27.8–60.4	1.64	1.2–2.2	0.17	0.05–0.5	75.3	62.8–85.3	76.2	48.9–93.4
>12.41	87.80	73.8–95.9	53.85	37.2–69.9	1.90	1.3–2.7	0.23	0.09–0.5	77.9	65.1–87.8	70.4	46.8–88.0
>13.22	85.37	70.8–94.4	66.67	49.8–80.9	2.56	1.6–4.1	0.22	0.1–0.5	82.6	69.7–91.6	71.0	50.1–87.0
>14.37	82.93	67.9–92.8	71.79	55.1–85.0	2.94	1.7–4.9	0.24	0.1–0.5	84.5	71.7–93.1	69.4	49.2–85.3
>15.15	82.93	67.9–92.8	76.92	60.7–88.9	3.59	2.0–6.5	0.22	0.1–0.4	87.0	74.3–94.9	70.8	51.4–85.9
>16.43	78.05	62.4–89.4	84.62	69.5–94.1	5.07	2.4–10.8	0.26	0.1–0.5	90.4	77.7–97.2	67.5	49.6–82.3
>17.60	78.05	62.4–89.4	87.18	72.6–95.7	6.09	2.6–14.0	0.25	0.1–0.5	91.9	79.6–98.0	68.1	50.3–82.8
>18.23	78.05	62.4–89.4	92.31	79.1–98.4	10.5	3.4–30.5	0.24	0.1–0.4	95.0	83.4–99.3	69.4	52.1–83.4
>19.13	75.61	59.7–87.6	94.87	82.7–99.4	14.4	3.8–57.5	0.26	0.1–0.4	96.5	85.3–99.8	67.7	50.8–81.7
>20.0	75.61	59.7–87.6	97.44	86.5–99.9	29.9	4.2–05.7	0.25	0.1–0.4	98.2	88.0–100.0	68.3	51.4–82.2
>21.88	63.41	46.9–77.9	100.00	91.0–100.0			0.37	0.2–0.5	100.0	89.1–100.0	59.5	44.2–73.6
>23.41	60.98	44.5–75.8	100.00	91.0–100.0			0.39	0.3–0.6	100.0	88.8–100.0	58.0	42.9–72.1
>26.42	53.66	37.4–69.3	100.00	91.0–100.0			0.46	0.3–0.6	100.0	87.2–100.0	53.7	39.4–67.7
>27.24	51.22	35.1–67.1	100.00	91.0–100.0			0.49	0.4–0.7	100.0	86.8–100.0	52.5	38.3–66.4
>28.42	46.34	30.7–62.6	100.00	91.0–100.0			0.54	0.4–0.7	100.0	85.8–100.0	50.1	36.3–63.9
>29.32	43.90	28.5–60.3	100.00	91.0–100.0			0.56	0.4–0.7	100.0	84.6–100.0	49.0	35.5–62.6
>29.77	41.46	26.3–57.9	100.00	91.0–100.0			0.59	0.5–0.8	100.0	83.9–100.0	47.9	34.6–61.4
>30.43	34.15	20.1–50.6	100.00	91.0–100.0			0.66	0.5–0.8	100.0	80.5–100.0	45.0	32.3–58.1
>32.94	19.51	8.8–34.9	100.00	91.0–100.0			0.80	0.7–0.9	100.0	69.2–100.0	40.1	28.5–52.6
>33.31	17.07	7.2–32.1	100.00	91.0–100.0			0.83	0.7–1.0	100.0	63.1–100.0	39.4	28.0–51.7
>34.39	14.63	5.6–29.2	100.00	91.0–100.0			0.85	0.8–1.0	100.0	59.0–100.0	38.7	27.4–50.9
>35.79	9.76	2.7–23.1	100.00	91.0–100.0			0.90	0.8–1.0	100.0	47.8–100.0	37.4	26.4–49.4
>36.36	7.32	1.5–19.9	100.00	91.0–100.0			0.93	0.9–1.0	100.0	29.2–100.0	36.7	26.0–48.6
>37.15	4.88	0.6–16.5	100.00	91.0–100.0			0.95	0.9–1.0	100.0	15.8–100.0	36.1	25.5–47.9
>39.69	2.44	0.06–12.9	100.00	91.0–100.0			0.98	0.9–1.0	100.0	2.5–100.0	35.6	25.0–47.2
>42.56	0.00	0.0–8.6	100.00	91.0–100.0			1.00	1.0–1.0			35.0	24.7–46.5
